# Supplementary material for: PRMT1-SFPQ regulates intron retention to control matrix gene expression during craniofacial development
Source: eLife. 2026 Sep 22;13:RP101386. doi: 10.7554/eLife.101386 (PMC13597084; doi:10.7554/eLife.101386)
Supplement: Supplementary file 1 [file elife-101386-fig1-data1.pdf]

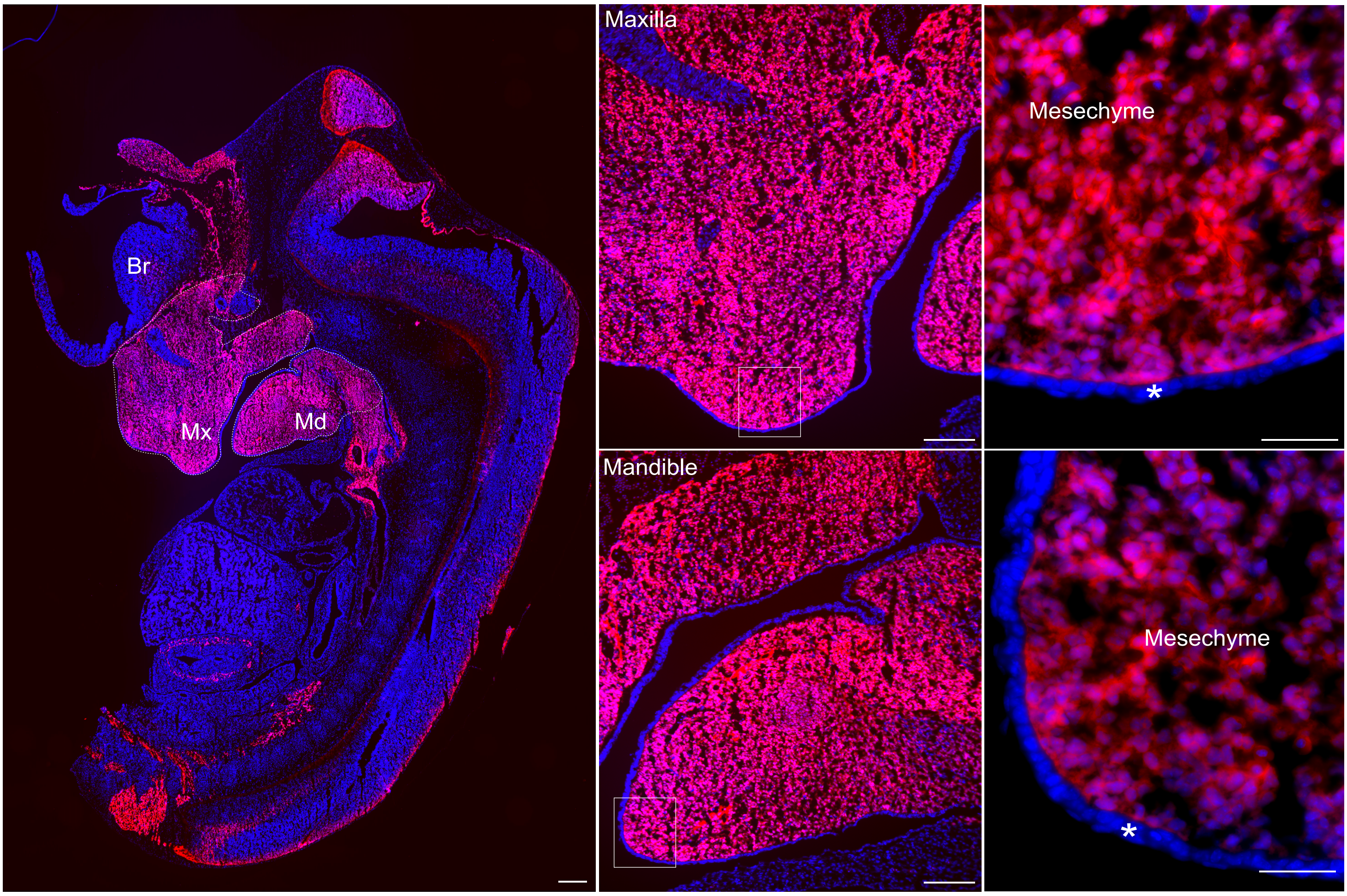


Br: Brain; Mx: Maxilla, Md: Mandible. * Epithelium.
