# Supplementary material for: PRMT1-SFPQ regulates intron retention to control matrix gene expression during craniofacial development
Source: eLife. 2026 Sep 22;13:RP101386. doi: 10.7554/eLife.101386 (PMC13597084; doi:10.7554/eLife.101386)
Supplement: Supplementary file 2 [file elife-101386-fig1-data3.pdf]

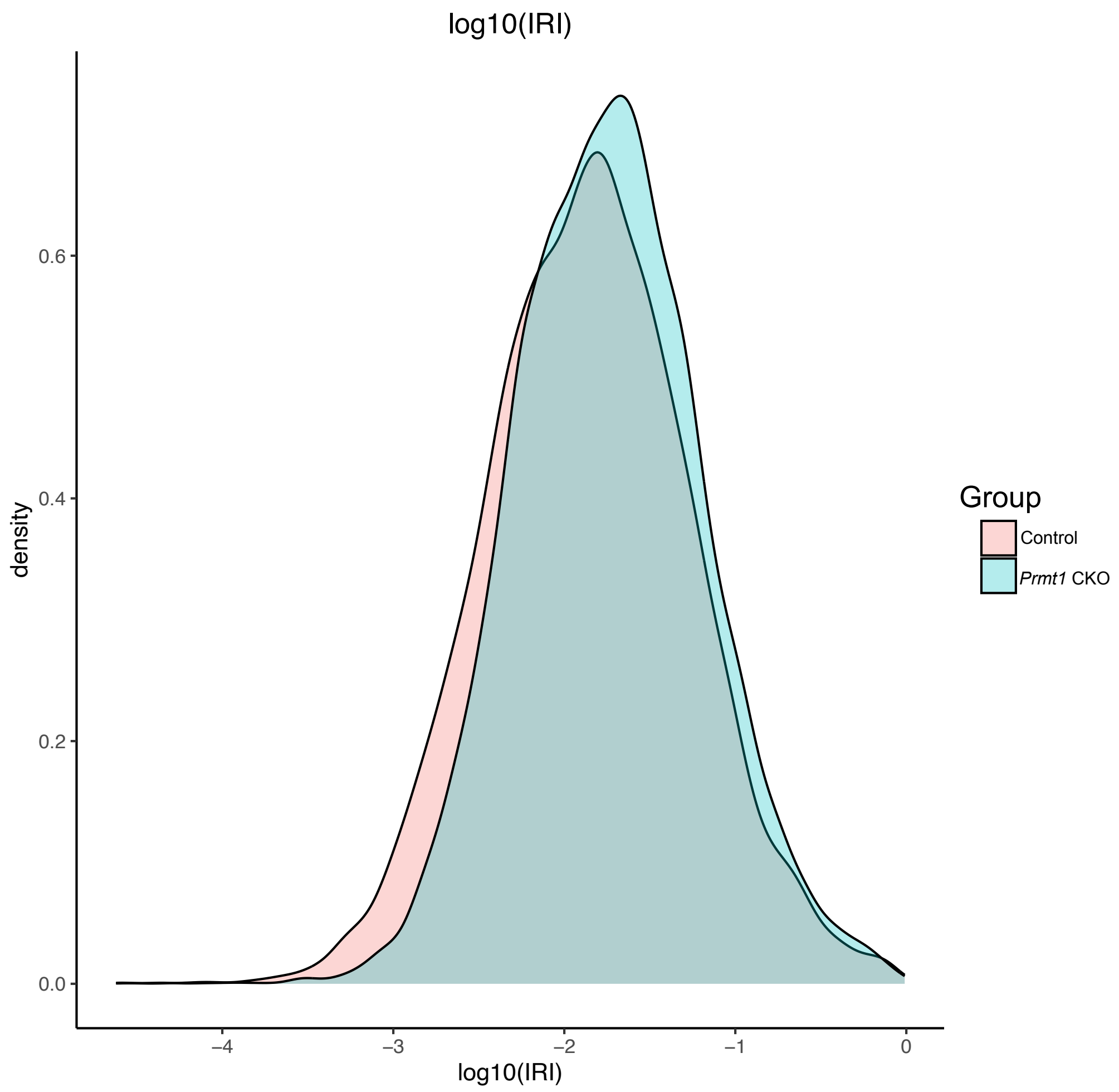
log10(IRI)
density
0.0
0.2
0.4
0.6
−4
−3
−2
−1
0
log10(IRI)
Group
Control
Prmt1 CKO
