# Supplementary material for: PRMT1-SFPQ regulates intron retention to control matrix gene expression during craniofacial development
Source: eLife. 2026 Sep 22;13:RP101386. doi: 10.7554/eLife.101386 (PMC13597084; doi:10.7554/eLife.101386)
Supplement: Supplementary file 5 [file elife-101386-fig5-data1.pdf]

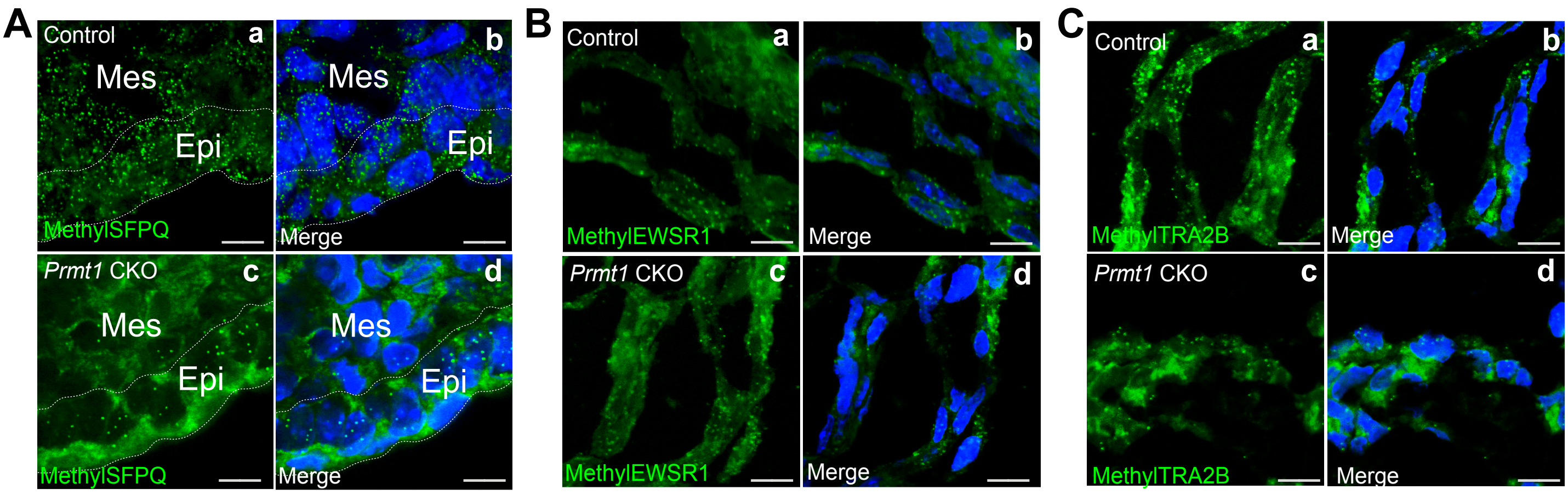
A
Control
a
Mes
Epi
MethylSFPQ
b
Mes
Epi
Merge
Prmt1 CKO
c
Mes
Epi
MethylSFPQ
d
Mes
Epi
Merge
B
Control
a
MethylEWSR1
b
Merge
Prmt1 CKO
c
MethylEWSR1
d
Merge
C
Control
a
MethylTRA2B
b
Merge
Prmt1 CKO
c
MethylTRA2B
d
Merge
