# Supplementary material for: PRMT1-SFPQ regulates intron retention to control matrix gene expression during craniofacial development
Source: eLife. 2026 Sep 22;13:RP101386. doi: 10.7554/eLife.101386 (PMC13597084; doi:10.7554/eLife.101386)
Supplement: Supplementary file 7 [file elife-101386-fig7-data1.pdf]

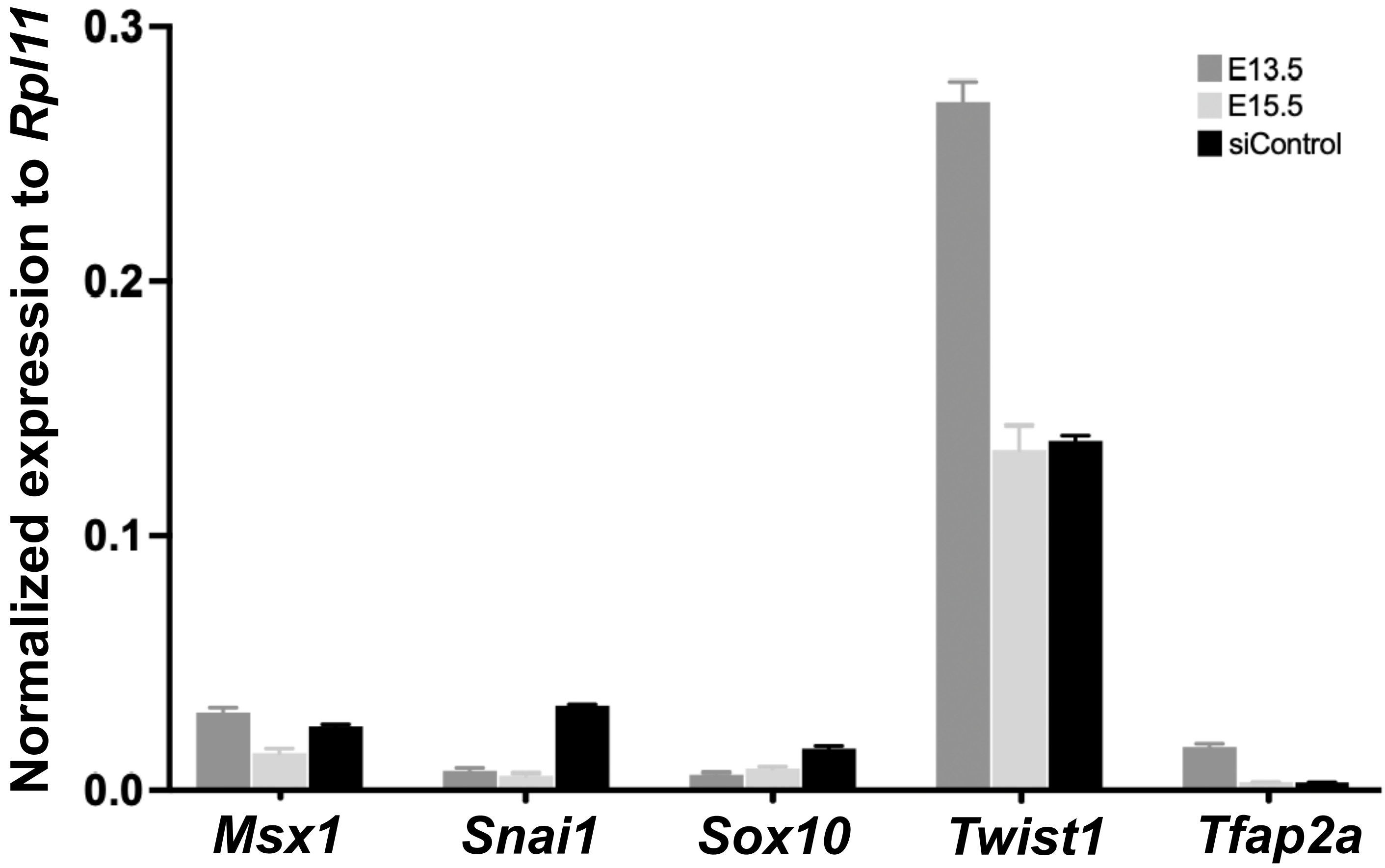
Normalized expression to Rpl11
0.3
0.2
0.1
0.0
E13.5
E15.5
siControl
Msx1
Snai1
Sox10
Twist1
Tfap2a
