# Supplementary material for: PRMT1-SFPQ regulates intron retention to control matrix gene expression during craniofacial development
Source: eLife. 2026 Sep 22;13:RP101386. doi: 10.7554/eLife.101386 (PMC13597084; doi:10.7554/eLife.101386)
Supplement: Peer review history

# Editor report: eLife Assessment

**DOI:** 10.7554/eLife.101386.3.sa0

**Authors:** Loydie A Jerome-Majewska

**Affiliations:** McGill University Canada

**Keywords:** Important, Solid

This **important** work establishes a connection between PRMT1 and SFPQ by identifying common phenotypes downstream of their inactivation. In the resubmission, authors now include NMD as a contributor to aberrant gene expression underpinning craniofacial development. The complementary experiments help strengthen some **solid** conclusions. This paper describes an interesting mechanism for the regulation of RNA levels, which is of interest to the readers of eLife.

# Referee report: Reviewer #1 (Public review)

**DOI:** 10.7554/eLife.101386.3.sa1

The current manuscript investigates a regulatory axis containing Prmt1, which methylates RNA binding proteins and alters intron splicing outcomes and expression of matrix genes. Authors test the effects of deficient Prmt1, Sfpq, and various other factors, using a combination of bioinformatic analyses and wet-lab validation approaches. Authors show that intron retention often triggers NMD, contributing to aberrant gene expression regulation and craniofacial development. The revised manuscript introduces several complementary experiments that help to strengthen conclusions. For example, authors directly investigate NMD-mediated transcript turnover to better understand how retention contributes to expression changes in genes of interest, and they assess several additional factors downstream of Prmt1 to justify a centralized interested in the PRMT1/SFPQ axis.

Weaknesses:

However, some points remain unaddressed or unexplored, which could bolster conclusions. For example, the transcriptome data from knockdown experiments indicate robust exon skipping, suggesting that analysis of these patterns in parallel with intron retention could provide additional insights into the responsive gene programs. Given that SFPQ is known to have multiple regulatory roles, a more thorough investigation of its possible mechanisms of action during craniofacial development would allow for definitive conclusions about the isolated impact of SFPQ-dependent splicing. Although authors employ CUT&Tag analysis of Pol II binding at the promoters and across the gene body, at the current scope, no change in Pol II association (i.e., absence of transcriptional repression) does not directly indicate a lack of transcriptional regulation by other means (pause release, elongation rate or processivity, transcription termination, etc.). Without a more thorough investigation of these mechanisms, this confounds definitive claims about their relative contributions to the gene expression landscape.

# Referee report: Reviewer #2 (Public review)

**DOI:** 10.7554/eLife.101386.3.sa2

Summary:

The manuscript by Lima et al examines the role of Prmt1 and SFPQ in craniofacial development. Specifically, the authors test the idea that Prmt1 directly methylates specific proteins that results in intron retention in matrix proteins. The protein SFPQ is methylated by Prmt1 and functions downstream to mediate Prmt1 activity. The genes with retained introns activate the NMD pathway to reduce the RNA levels. This paper describes an interesting mechanism for the regulation of RNA levels during development.

Strengths:

The phenotypes support what the authors claim that Prmt1 is involved in craniofacial development and splicing. They use of state of the art sequencing to determine the specific genes that have intron retention and changes in gene expression is a strength.

Weaknesses:

The results now support the conclusions;however, it is still unclear how direct the relationship is between Prmt1 and SFPQ.

# Author comment: Author response

**DOI:** 10.7554/eLife.101386.3.sa3

**Authors:** Julia Raulino Lima, Nicha Ungvijanpunya, Qing Chen, Hoang Quoc Hai Pham, Tal Rosen, Greg Park, Mohammadreza Vantankhah, Steven Yen, Yang Chai, Amy E Merrill, Zhaoyang Liu, Jianfu Chen, Yanzhong Yang, Weiqun Peng, Jian Xu

**Affiliations:** University of Southern California Los Angeles United States; University of Southern California Los Angeles United States; The George Washington University Washington DC United States; City Of Hope National Medical Center Duarte United States; University of Southern California Los Angeles United States; University of Southern California Los Angeles United States; University of Southern California Los Angeles United States; University of Southern California Los Angeles United States; University of Southern California Los Angeles United States; University of Southern California Los Angeles United States; University of Southern California Los Angeles United States; University of Southern California Los Angeles United States; City Of Hope National Medical Center Duarte United States; The George Washington University Washington United States; University of Southern California Los Angeles United States

The following is the authors’ response to the original reviews.

**Public Reviews:**

**Reviewer #1 (Public review):**

The strength of the current study lies in their establishing the molecular mechanism through which PRMT1 could alter craniofacial development through regulation of the transcriptome, but the data presented to support the claim that a PRMT1-SFPQ axis directly regulates intron retention of the relevant gene networks should be robust and with multiple forms of clear validation. For example, elevated intron retention findings are based on the intron retention index, and according to the manuscript, are assessed considering the relative expression of exons and introns from a given transcript. However, delineating between intron retention and other forms of alternative splicing (i.e., cryptic splice site recognition) requires a more comprehensive consideration of the intron splicing defects that could be represented in data. A certain threshold of intron read coverage (i.e., the percent of an intron that is covered by mapped reads) is needed to ascertain if those that are proximal to exons could represent alternative introns ends rather than full intron retention events. In other words, intron retention is a type of alternative splicing that can be difficult to analyze in isolation given the confounding influence of cryptic splicing and cryptic exon inclusion. If other forms of alternative splicing were assessed and not detected, more confident retention calls can be made.

This manuscript is a mechanistic exploration that follows previous work we published on the role of *Prmt1* in craniofacial development, in which genetic deletion of *Prmt1* in CNCCs leads to cleft palate and mandibular hypoplasia (PMID: 29986157).

As the reviewer pointed out, a certain threshold of intron read coverage is needed to assess intron retention events. We employed IRTools to assess the collective changes of intron retention between cell-states associated with certain biological function or pathway. IRTools incorporated considerations for intron read coverage by checking the evenness of read distribution in an intron. Specifically, every constitutive intronic regions (CIR) is divided into 10 equally sized bins and the proportion of reads that map to each bin is calculated. CIRs are then ranked according to their imbalance in bin-wise reads distribution, represented by the proportion of reads in its most populated bin. Those among top 1% are considered to contain potentially false IR events and excluded. We further addressed this question by developing another measure of intron retention, intron retention coefficient (IRC), which assesses IR events using the junction reads (Supplemental Figure-S8). Junction reads that straddle two exons are called exon-exon junction reads (spliced reads), and those that straddle an exon and a neighboring intron are called exon-intron junction reads (retained reads). The IRC of an intron is defined as the fraction of junction reads that are exon-intron junction reads: IRC = exon-intron read-count / (exon-exon read-count + exon-intron read-count), where exon-intron read-count = (5’ exon-intron read-count + 3’ exon-intron read-count) / 2. The IRC of a gene is defined as the exon-intron fraction of all junction reads overlapping or over the constitutive introns of this gene. In the calculation of the IRC, only exon-intron junction reads that cover the junction point and overlap both of each side for at least 8 bps were counted, and only exon-exon junction reads that jump over the relevant junction points and overlap each of the respective exons for at least 8 bps were counted. In this process, evenness of the proportion of exon-intron junction reads that are 5’ or 3’ exon-intron junction reads are taken into account. As shown in the Supplemental Figure S7A and S7B, IRC analysis generated consistent results with those obtained from using IRI (Figure 3A and 3I).

In addition, as the reviewer pointed out, intron retention can be difficult to analyze in isolation. We followed the reviewer’s suggestion that “If other forms of alternative splicing were assessed and not detected, more confident retention calls can be made“ and analyzed other forms of alternative splicing for all *ECM* and *GAG* genes with significant IRI increase (genes highlighted in Figure-3A and 3I) using rMATS (Supplemental Figure-S9). Among these genes, only 5 genes (*Cthcr1, Mmp23, Adamts10, Ccdc80* and *Col25a1*) showed statistically significant changes in skipped exon, 1 gene (*Bmp7*) showed significant changes in mutually exclusive exons, and none showed significant changes in alternative 5’ or 3’ splicing. SE and MXE changes detected were marginal (Supplemental figure S8), while the majority of matrix genes with significant intron retention didn’t exhibit other forms of alternative splicing, further supporting the confidence of intron retention calls.

While data presented to support the PRMT1-SFPQ activation axis is quite compelling, that this is directly responsible for the elevated intron retention remains enigmatic. First, in characterizing their PRMT1 knockout model, it is unclear whether the elevated intron retention events directly correspond to downregulated genes.

In the revised manuscript, we demonstrate IR-triggered NMD as a mechanism for transcript decay and downregulation of matrix genes. When IR-triggered NMD was blocked by chemical inhibitor NMDI14, the intron-retaining transcripts showed significant accumulation (new Figure-4). NMD is the RNA surveillance system to degrade aberrant RNAs. Intron retention-triggered NMD in cancer has both promotive and suppressive roles and NMD inhibitors has been tested for cancer therapy including immunotherapy. During embryonic development, the functional significance of NMD machinery is suggested by human genetic findings and mouse genetic models. NMD is driven by a protein complex composed of SMG and UPF proteins. *Smg6, Upf1, Upf2* and *Upf3a* knockout mouse die at early embryonic stages (E5.5-E9.5), and *Smg1* gene trap mutant mice die at E12.5 (PMID: 29272451). *SMG9* mutation in human patients causes malformation in the face, hand, heart and brain (PMID: 27018474).

We show that in CNCCs NMD functions both as a physiological mechanism and invoked by molecular insult. Blocking NMD in CNCCs caused significant accumulation of intron-retaining *Adamts2, Alpl, Eln, Matn2, Loxl1* and *Bgn* transcripts, suggesting a basal role for NMD to degrade intron-retaining transcripts (Figure-4Ba-4Bf). We further demonstrated the accumulation of *Adamts2* and *Fbln5* using semi-quantitative PCR with the detection of a longer product from *Adamts2 intron 19* and *Fbln5 intron 7* (Figure-4Ca-4Ch). In CNCCs and ST2 cells, NMD is further invoked by *Prmt1* and *Sfpq* deficiency. In *Prmt1* deficient CNCCs, NMD blockage led to higher accumulation of intron-retaining *Adamts2* and *Alpl* transcripts, suggesting that *Prmt1* deficiency triggers NMD to reduce intron-containing transcripts (Figure-4Aa, 4Ab). In *Sfpq*-depleted ST2 cells, blocking NMD caused accumulation of intron-retaining transcripts *Col4a2, St6galnac3* and *Ptk7* (Figure-9B, 9C).

Moreover, intron splicing is a well-documented node for gene regulation during embryogenesis and in other proliferation models, and craniofacial defects are known to be associated with 'spliceosomopathies'. However, reproduction of this phenotype does not suggest that the targets of interest are inherently splicing factors, and a more robust assessment is needed to determine the exact nature of alternative splicing in this system. Because there are several known splicing factors downstream of PRMT1 and presented in the supplemental data, the specific attribution of retention to SFPQ would be additionally served by separating its splicing footprint from that of other factors that are primed to cause alternative splicing.

We have previously shown that a group of splicing factors depends on *Prmt1* for arginine methylation, including SFPQ (PMID: 31451547). We tested additional splicing factors that are highly expressed in CNCCs and depends on PRMT1 for arginine methylation: SRSF1, EWSR1, TAF15, TRA2B and G3BP1 (Figure-5, 6 and 10). Among these factors, EWSR1 and TRA2B are both methylated in CNCCs and depend on PRMT1 for methylation (Fig. 5 and Supplemental Figure-S3B, S3C). We weren’t able to assess TAF15 methylation because of lack of efficient antibody for the PLA assay. We also demonstrated that their protein expression or subcellular localization was not altered by *Prmt1* deletion in CNCCs, unlike SFPQ (Supplemental Figure-S4). To define their splicing footprint, we performed siRNA-mediated knockdown in ST2 cells, followed by RNA-seq and IRI analysis to define differentially regulated genes and introns, which revealed distinct biological pathways regulated by SFPQ, EWSR1, TRA2B and TAF15, but minimal roles of EWSR1, TRA2B and TAF15 on intron retention when compared to SFPQ (Fig. 10F-10S, Supplemental Figure S7A-S7F, Supplemental Tables S4-S6). ECM genes are significantly downregulated by all four splicing factors (Fig. 10F-10I), but EWSR1, TRA2B and TAF15 function through IR-independent mechanisms, such as exon skipping, as exemplified by *Postn* (Fig. 10J-10S).

Clarifying the relationship between SFPQ and splicing regulation is important given that the observed splicing defects are incongruous with published data presented by Takeuchi et al., (2018) regarding SFPQ control of neuronal apoptosis in mice. In this system, SFPQ was more specifically attributed to the regulation of transcription elongation over long introns and its knockout did not result in significant splicing changes. Thus, to establish the specificity for the SFPQ in regulating these retention events, authors would need to show that the same phenotype is not achieved by mis-regulation of other splicing factors. That the authors chose SFPQ based on its binding profile is understandable but potentially confounding given its mechanism of action in transcription of long introns (Takeuchi 2018). Because mechanisms and rates of transcription can influence splicing and exon definition interactions, the role of SFPQ as a transcription elongation factor versus a splicing factor is inadequately disentangled by authors.

To test whether SFPQ acts as a transcription elongation factor, we performed Pol II Cut&Tag in ST2 cells and demonstrated that depletion of SFPQ only caused marginal changes in either the promoter region or gene body of ECM genes, suggesting that the role of SFPQ as a transcriptional activator or elongation factor is minimal (Fig. 7G, 7H). This finding is distinct from SFPQ function in neurons (PMID: 29719248), suggesting that the activation or recruitment of SFPQ in transcriptional regulation may involve tissue-specific factors in neurons.

**Reviewer #2 (Public review):**

Summary:

The manuscript by Lima et al examines the role of Prmt1 and SFPQ in craniofacial development. Specifically, the authors test the idea that Prmt1 directly methylates specific proteins that results in intron retention in matrix proteins. The protein SFPQ is methylated by Prmt1 and functions downstream to mediate Prmt1 activity. The genes with retained introns activate the NMD pathway to reduce the RNA levels. This paper describes an interesting mechanism for the regulation of RNA levels during development.

Strengths:

The phenotypes support what the authors claim that Prmt1 is involved in craniofacial development and splicing. The use of state-of-the-art sequencing to determine the specific genes that have intron retention and changes in gene expression is a strength.

Weaknesses:

Some of the data seems to contradict the conclusions. And it is unclear how direct the relationships are between Prmt1 and SFPQ.

**Recommendations for the authors:**

**Reviewer #1 (Recommendations for the authors):**

First, the claims regarding the effect of PRMT1 loss on splicing are unclear by the section title. In other words, does loss PRMT1 change the incidence of baseline alternative splicing events, or does it introduce new retention events that are responsible for underwriting the craniofacial phenotype? Consistent with this idea, the narrative could benefit from more cellular and/or histological validations of the transcriptomic defects discovered in the RNAseq, which could help contextualize the bioinformatics data with the developmental defects. Moreover, the conclusions drawn about intron retention could be clarified in terms of how applicable the mechanism is likely to be outside of this tissue-specific set of responsive introns.

Loss of *Prmt1* did not cause a global shift in intron retention, as shown in Supplemental Figure S2. Instead, *Prmt1* deletion caused increase of intron retention specifically in genes enriched in cartilage development, glycosaminoglycan biology, dendrite and axon, and decreased intron retention in mitochondria and metabolism genes (Table. S1). We also tested matrix protein expression by histology to confirm that transcriptomic defects revealed at the RNA level resulted in lower protein production. The new data are in Figure 3E-3H.

Additionally, invoking NMD to align splicing and differential gene expression data understandable but lacking sufficient controls to be conclusive, such as positive control genes to confirm inhibition of NMD.

To validate the blockage of NMD, *glutathione peroxidase 1 (Gpx1) intron 1*, a well-documented substrate for NMD, is tested as positive control (Fig 4Ac, 4Ad, 9B).

Additionally, it should be clarified whether NMD is a basal mechanism for the regulation of these introns or whether it is an induced mechanism that is invoked by the molecular insult.

In CNCCs, NMD functions both as a physiological mechanism and invoked by molecular insult. Please refer to responses to Reviewer 1’s public review for detailed explanations.

Further, authors present data downstream of two siRNAs for the same gene target, but it remains unclear how siRNAs for the same gene target produce different effects. It may be helpful for authors to clarify how many of the transcriptomic defects are shared versus unique between the siRNAs.

To address this question, we used bioinformatic analysis of the whole genome data to the similarity in changes caused by the two SFPQ-targeting siRNAs. As shown in the new Fig. 7Ba & 7Bb, transcriptomic and intron changes are consistent between the two siRNAs, suggesting that genes targeted by the two siRNA predominantly overlap. This overlap is illustrated by scatter plot analysis of RNAseq DEG and IRI data from each siRNA against SFPQ.

Finally, we stress the importance of presenting the full conceptual basis for SFPQ's potential role in splicing and gene expression. It is significant to note that SFPQ has been previously studied as a splicing factor and was instead determined to function in support of the transcription elongation rather than in splicing. Thus, if authors are confident that the SFPQ manifests directly in splicing changes they encumber the burden of proof to show that its role in transcription, nor another splicing factor, are driving splicing changes.

We demonstrated that depletion of SFPQ only caused marginal changes in either the promoter region or gene body of *ECM* genes, suggesting that the role of SFPQ as a transcriptional activator or elongation factor is minimal (Fig. 7G, 7H). Please refer to responses to Reviewer 1’s public review for detailed explanations.

**Reviewer #2 (Recommendations for the authors):**

(1) It is not clear why the authors focused on intron retention targets vs the other possibilities. Skipped Exon is much higher in terms of the number of changes, please clarify. For the intron retention how is this quantified? The traces are nice, but it is hard to tell which part is retained at this magnification. Also, because the focus is on extracellular matrix (ECM) and NMD it would be nice to show some of those targets here. In the tbx1 trace, some are up and some are down. What does that mean for the gene expression?

We have investigated SE initially and found that genes with significant changes in *Prmt1* CKO CNCCs fall into diverse functional pathways. Among them, a few genes are critical for skeletal formation, including *Postn* and *Fn*, and the function of their exon skipping has been documented. For example, the two exons that are skipped in *Postn, Exon17* and 21, have been shown to regulate craniofacial skeleton shape and mandibular condyle hypertrophic zone thickness using transgenic mouse models (PMID: 36859617). As illustrated by Figure 10, the skipped exon of *Postn* is regulated by multiple splicing factors that may perform overlapping functions in vivo.

Intron retention of each gene is quantified by the ratio of the overall read density of its constitutive intronic regions (CIRs) to the overall read density of its constitutive exonic regions (CERs) and defined as the intron retention index (IRI). In the first section of Response to Reviewer 1’s comments, we explained additional bioinformatic analysis that was performed to address reviewers’ questions, support the confidence of intron event calls and rule out the possibility of other alternative splicing mechanisms, such as by SE, MXE, A5SS or A3SS (Supplemental Figure S5, S6, Table S7).

(2) RNA-Sequencing of Prmt1 mutants nicely shows gene expression changes, including in ECM and GAG genes. While validation of the sequencing results is not necessarily required, it would be very interesting to show the expression in situ. In addition, the heat map shows both downregulated but also upregulated transcripts. This is expected since this protein regulates many genes. However, the volcano plot shows a significant number of genes upregulated. It would be interesting to show what the upregulated genes are. And what is the proposed mechanism for Prmt1 regulation of upregulated genes?

Validation for the transcriptomic changes is shown in Fig. 3E-3H using immunostaining.

As for upregulated genes in *Prmt1* mutant, top pathways include cytokine-mediated signaling pathway, signal transduction by p53 signaling pathway and cell morphogenesis (Figure 2E), which are consistent with our previous reports that Prmt1 deletion induces cytokine production in oral epithelium and leads to p53 accumulation in embryonic epicardium (PMID: 32521264, 29420098). Besides these pathways, *Prmt1* deletion also caused upregulation of genes involved in adult behavior, postsynaptic organization and apoptotic process, which is consistent with findings from other labs on PRMT1 function in neuronal and cancer cells (PMID: 34619150, 33127433).

(3) Specific transcripts were shown to have elevated intron retention involved in the ECM and GAG pathway. However in Figure 3D it seems to show the opposite with intronic expression decreased and exonic increases and intronic decrease. This is very important to the final conclusion of the paper. In addition, is there a direct relationship between increased intron and downregulation of this specific gene expression? It seems a bit correlational as it could also be an indirect mechanism. One way to test this is to do in vitro translation with and without the specific intron to test if it results in lower expression.

We apologize for the mis-labeling in previous version of Figure 3D, which is now corrected. We also tried to test the direct relationship between intron and downregulation of matrix genes such as *Adamts2* using in vitro experiments, however, the introns of matrix genes with high retention tends to be long, many 10 to 50kb in length, making it challenging to generate mini-gene constructs for molecular analysis. We used a different approach and demonstrated that inhibition of NMD with a chemical inhibitor NMDI14 caused dramatic accumulation of the *Adamts2, Alpl, Eln, Matn2, Loxl1* and *Bgn* transcripts, suggesting that retained introns triggered NMD to regulate gene expression and this mechanism acts as a physiological level in CNCCs (Fig. 4). We also blocked NMD in control and *Prmt1* null CNCCs, where NMD blockage led to higher accumulation of *Adamts2* and *Alpl* transcripts, suggesting that upon *Prmt1* deficiency, NMD is further utilized to degrade intron-containing transcripts (Fig. 4). Similarly, in *Sfpq*-depleted ST2 cells, blocking NMD caused accumulation of intron-retaining transcripts *Col4a2, St6galnac3* and *Ptk7* (Fig. 9A, 9B).

(4) While Figure 4 nicely shows the methylation of SFPQ is reduced in Prmt1 CKO cells, it is unclear which reside this methylation occurs. Also the overall expression of SFPQ is also down so it is possible that the methylation is indirect ie Prmt1 regulates some other methyltransferase that regulates SFPQ. Or that because the overall level of SFPQ is down, there is no protein to methylate. How do the authors differentiate between these possibilities?

Previously, arginine methylation of SFPQ has been characterized using in vitro reaction and cell lines with biochemical assays by Snijders., et al in 2015 (PMID: 25605962). Among all PRMTs that catalyze asymmetric arginine dimethylation (ADMA), SFPQ is methylated by only PRMT1 and PRMT3, with PRMT1 showing higher efficiency while PRMT3 showing a lower efficiency. However, PRMT3 is mainly cytosolic. Its expression in CNCCs is about 100-fold lower than PRMT1 (Fig. 1). Based on these knowledges, PRMT1 is the primary arginine methyltransferase for SFPQ, a nuclear protein in CNCCs. We and others have shown in a previous publication that SFPQ methylation on arginine 7 and 9 depends on PRMT1 (PMID: 31451547).

To investigate SFPQ protein degradation in CNCCs, we used MG132 to block proteasomal degradation and observed a partial rescue of SFPQ protein degradation in Prmt1 mutant embryos, suggesting that SFPQ is degraded through proteasomal-mediated mechanism. To address the relationship between SFPQ methylation and protein expression, we assessed arginine methylation of SFPQ that accumulated after MG132 treatment. The accumulated SFPQ was not methylated, confirming the absence of methylation even when SFPQ protein expression is restored.

Snijders., et al, also shown that citrullination induced by PADI4 regulate SFPQ stability (Snijders 2015). We considered this possibility and assessed the expression levels of PADIs. In E13.5 and E15.5 CNCCs, PADI1-4 mRNA expression levels are very low (TPM<5), suggesting that PADIs may not regulate SFPQ stability in CNCCs. A detailed mechanism as to how PRMT1-mediated SFPQ methylation controls stability awaits further investigation.

(5) For the Sfpq deleted experiment, it seems that the two knockdowns are not similar in the gene targets and GO terms different except Wnt signaling. This makes this data difficult to interpret. The genes identified as intron retention are different than the ones identified in Prmt1 deletion and not reduced as much. How does this fit in with the Prmt1 story? If working through Sfpq, it assumes that the targets will be similar and more the 8% would be in common.

To address the first concern, we used bioinformatic analysis of the whole genome data to the similarity in changes caused by the two SFPQ-targeting siRNAs. As shown in the new Fig. 7Ba & 7Bb, transcriptomic and intron changes are consistent between the two siRNAs, suggesting that genes targeted by the two siRNA predominantly overlap. This overlap is illustrated by scatter plot analysis of RNAseq DEG and IRI data from each siRNA against SFPQ.

We have previously identified a group of splicing factors that depends on PRMT1 for arginine methylation, including SFPQ (PMID: 31451547). In the new data in Figures 5, 6 and 10, we tested an additional five PRMT1-dependent splicing factors that are highly expressed in CNCCs: SRSF1, EWSR1, TAF15, TRA2B and G3BP1 (Fig. 5, 6 and 10). Among these factors, SRSF1 and G3BP1 are predominantly expressed in the cytosol of NCCs at E13.5. As splicing activity in the nucleus is needed for pre-mRNA splicing, we excluded these two and focused on the other three proteins. EWSR1 and TRA2B are both methylated in CNCCs and depend on PRMT1 for methylation (Fig. 5). We weren’t able to assess TAF15 methylation because of lack of efficient antibody for the PLA assay. We also demonstrated that their protein expression or subcellular localization was not altered by *Prmt1* deletion in CNCCs, unlike SFPQ (Fig. S2). To define their splicing footprint, we performed siRNA-mediated knockdown in ST2 cells, followed by RNA-seq and IRI analysis to define differentially regulated genes and introns, which revealed distinct biological pathways regulated by SFPQ, EWSR1, TRA2B and TAF15, but minimal roles of EWSR1, TRA2B and TAF15 on intron retention when compared to SFPQ (Fig. 10F-10I, Supplemental Figure S7A-S7F). ECM genes are significantly downregulated by all four splicing factors (Fig. 10J-10M), but EWSR1, TRA2B and TAF15 regulate transcription or exon skipping instead of IR, as exemplified by *Alpl* and *Postn* (Fig. 10N-10T).

(6) The addition of an NMD mechanism is interesting but not surprising that when inhibiting the pathway broadly, there is an increase in gene expression in the mesoderm cell line. How specific is this to craniofacial development?

NMD is driven by a protein complex composed of SMG and UPF proteins. We show in the revised manuscript that NMD is both a physiological mechanism in CNCCs and triggered by genetic disturbance (Fig. 4). These data are in line with human patient reports where *SMG9* mutation in human causes malformation in the face, hand, heart and brain (PMID: 27018474). Mouse genetic studies also demonstrated roles of NMD components during embryonic development.*Smg6, Upf1, Upf2* and *Upf3a* knockout mouse die at early embryonic stages (E5.5-E9.5), and *Smg1* gene trap mutant mice die at E12.5 (Han 2018). Additionally, intron retention-triggered NMD in cancer has both promotive and suppressive roles and NMD inhibitors has been tested for cancer therapy and recently cancer immunotherapy. Our findings highlight matrix genes as one of the key targets for NMD during craniofacial development.

Minor:

(1) The supplemental figures are difficult to understand. In the first upload there are many figures and tables, some excel files that are separate uploads and some not. Please upload as separate files so it is clear. And also put them in order that they are in the manuscript.

(2) For the heat map in figure 2B, it would be good to show all the genes or none at all. It seems a bit like cherry-picking to highly only a few. And they are not labeled where they are located in the graph. Are these the top lines if so please label.

(3) Gene names in Figure 3A are difficult to read. I would also not consider BMP7 an ECM gene.

(4) A summary diagram of the interactions proposed will help to make this more understandable.

The supplemental figures are reorganized and uploaded as separate word and excel documents. For Heat map in Fig. 2B, we have removed the gene names. For Fig. 3A, only the most significantly changed gene are labeled in red dots with names. We didn’t label all the genes because of the large number of genes. For the new Figure 3B, we have replaced BMP7. A schematic summary is also added to Supplemental Fig. S9 to illustrate the PRMT1-SFPQ pathway.
